# Concrete by Preplaced Aggregate Method Using Silica Fume and Polypropylene Fibres

**DOI:** 10.3390/ma15061997

**Published:** 2022-03-08

**Authors:** Farooq Azam Khanzada, Kashif Nazir, Muhammad Ishtiaq, Muhammad Faisal Javed, Sardar Kashif-ur-Rehman, Fahid Aslam, Muhammad Ali Musarat, Kseniia Iurevna Usanova

**Affiliations:** 1 Department of Transportation Engineering (SCEE), National University of Sciences and Technology, Islamabad 44000, Pakistan; fazam111@gmail.com; 2 Department of Civil Engineering, School of Engineering, Nazabayev University, Astana 010000, Kazakhstan; kashif.nazir@nu.edu.kz; 3 Department of Civil Engineering, Capital University of Science & Technology, Islamabad 44000, Pakistan; ishtiaqm298@gmail.com; 4 Department of Civil Engineering, COMSATS University Islamabad, Abbottabad Campus, Abbottabad 22060, Pakistan; skashif@cuiatd.edu.pk; 5 Department of Civil Engineering, College of Engineering in Al-Kharj, Prince Sattam Bin Abdulaziz University, Al-Kharj 11942, Saudi Arabia; f.aslam@psau.edu.sa; 6 Department of Civil and Environmental Engineering, Universiti Teknologi PETRONAS, Bandar Seri Iskandar 32610, Perak, Malaysia; muhammad_19000316@utp.edu.my; 7 Peter the Great St. Petersburg Polytechnic University, 195291 St. Petersburg, Russia; plml@mail.ru

**Keywords:** preplaced aggregate, silica fume, polypropylene fibres, concrete, mass loss

## Abstract

Preplaced aggregate concrete (PAC) is prepared in two steps, with the coarse aggregate being initially laid down in the formwork, after which a specialised grout is injected into it. To enhance the properties of concrete and to reduce the emission of CO_2_ produced during the production of cement, supplementary cementitious materials (SCMs) are used to partially substitute ordinary Portland cement (OPC). In this study, 100 mm × 200 mm (diameter x height) PAC cylinders were cast with 10 per cent of cement being substituted with silica fume; along with that, 1.5% dosage of Macro polypropylene fibres were also introduced into the coarse aggregate matrix. Compressive strength test, splitting tensile strength test, mass loss at 250 °C, and compressive strength at 250 °C were performed on the samples. PAC samples with 10% of cement replaced with Silica Fume (SPAC) were used as control samples. The primary objective of this study was to observe the effect of the addition of Polypropylene fibres to PAC having Silica fume as SCM (FRPAC). The aforementioned tests showed that FRPAC had a lower compressive strength than that of the control mix (SPAC). FRPAC had greater tensile strength than that of NPAC and SPAC. Mass loss at 250 °C was greater in SPAC compared to FRPAC. The compressive strength loss at 250 °C was significantly greater in FRPAC compared to SPAC. FRPAC exhibited a greater strain for the applied stress, and their stress-strain curve showed that FRPAC was more ductile than SPAC.

## 1. Introduction

First introduced in the 1930s, preplaced aggregate concrete (PAC), otherwise called two-stage concrete [1,2], as the name suggests, is prepared in two steps. Initially, clean, gap-graded coarse aggregate is packed within the formwork then, a specialised grout of sufficient flowability is used to fill the openings and voids in the coarse aggregate matrix. Usually, admixtures are added to improve the properties of PAC, especially workability [3,4]. On the contrary, conventional concrete is prepared by mixing all the components and then placing that mix in the formwork. PAC has greater coarse aggregate content, accounting for 60% to 70% of the total volume. While in conventional concrete, the coarse aggregate content takes up only 40% to 50% of the total volume [5,6]. Therefore, the shrinkage in PAC is far less than that of conventional concrete [7]. Furthermore, PAC has a more dense matrix having greater strength as a result of higher pressure and point-to-point contact of the coarse aggregate particles [8]. Along with that, PAC’s production cost is 25% to 40% less than that of conventional concrete [3].

PAC has several uses, which include the construction of concrete structures underwater [4], repair work of pre-built concrete structures [9], mass concreting [10], construction of nuclear power plants [7], and structures involving complex reinforcement [9]. Since its development, PAC has been used in numerous projects throughout the world, for example, in the Pre-facing of Barker Dam at Nederland, Colorado in 1946 [11], in the Scroll case at Bull Dam Powerhouse in 1951 [7], and in Auxiliary dam located in China in 2006 [12]. As PAC has more coarse aggregate and lesser cement content, the demand for cement may be reduced. Moreover, the heat of PAC’s hydration is less than conventional concrete [10]. Therefore, PAC might be considered an eco-friendly alternative to conventional concrete. Another way of further reducing the cement content within PAC is by utilising waste supplementary cementitious materials (SCMs). They improve the durability and mechanical properties of concrete [13]. Efficient use of these SCMs may not only prove to be more eco-friendly and more economical but the carbon footprint of concrete would also be reduced [5]. SCMs include Silica fume (SF) and ground granulated blast furnace slag (GGBFS) [3].

Silica fume is a very fine and highly pozzolanic material with high silica content. Partial substitution of cement with SF has shown enhanced durability, chemical properties, and strength, but the mix’s workability was reduced [5,14]. Silica fume plays the role of a micro filler between the fine aggregate and cement particles, meanwhile increasing the water demand because of its very large surface area, subsequently causing the workability of the grout to decrease [14]. Silica fume reacts with Calcium Hydroxide, leading to greater amounts of Calcium Silicate Hydrate, which is the key factor in the strength development of concrete [13].

Concrete, which mainly suffers from lower tensile strength and higher rigidity, is termed as a brittle material [15,16,17]. However, higher strength and better durability are essential for the aforementioned uses of PAC; for this purpose, additional components are added to PAC to enhance its properties, especially strength and durability. One of the possible components to enhance the properties of concrete are fibres, which may be added in varying dosages. Fibres are used to replace the conventional reinforcement in concrete, either partially or completely, for example, in road pavements, slabs, beams, industrial floors, and tunnel linings [18,19]. The single critical shear crack formation could be avoided, and the occurrence of the shear failure mechanism could be delayed with the inclusion of fibres [20]. Fibres are especially useful in the structural members in which the installation of transverse reinforcements is either difficult or impossible [21,22]. A major benefit of using fibres in concrete is that it increases the toughness of the concrete; subject to any type of loading, the strain corresponding to the peak load is increased, and higher energy absorption is observed in the post-peak portion of the load-deflection curve [23]. Steel fibres are most commonly used [18], accounting for almost half of the total tonnage used [24]. Steel fibres increase the pre and post-cracking tensile strength of the concrete and allow for enhanced crack control [25]. Post-failure inspection of steel-fibre-reinforced concrete shows that the cracking pattern is more widely distributed, and there is a lesser separation between cracks [26]. Various researchers have undergone several studies to enhance the ductility of concrete by using steel fibres [27]. Steel fibres enhance the flexural strength, compressive strength, and modulus of elasticity of concrete [24]. Another type of fibre that may be used is Basalt fibre, obtained from Basalt rocks melted at 1400 °C. These fibres are non-toxic, environmentally friendly, and have high insulating properties [28,29]. The inclusion of Basalt fibres slightly improved the compressive strength of concrete at 28 days of curing. It also enhanced the concrete’s modulus of rupture and flexural strength [30]. Polyethylene terephthalate (PET) strings, a polyester polymer [31], are industrially cut, forming short, multifilament fibres, with a diameter of 30 micrometres, to be used in concrete for the sustainable development of concrete structures [32]. PET fibres enhance the ductility, tensile and compressive strengths, and thermal resistance of concrete [33]. Glass fibres reduce the fresh density of concrete and increase the split tensile strength, compressive strength, flexural strength, porosity, and chloride penetration of concrete [34]. Flax fibres, a bio-based material obtained from plants due to their environmentally friendly nature, are used in concrete for the sustainable development of the concrete industry [35]. These fibres tend to decrease the workability and compressive strength of the concrete. The inclusion of flax fibres in concrete leads to an increase in the air content of fresh concrete, whereas porosity and flexural strength of the concrete also increase [36].

According to previous studies, on the addition of Macro Polypropylene fibres (PP) to conventional concrete, its flexural strength, ductility, and durability were improved, while brittle failure, corrosion of steel reinforcement, plastic, and drying shrinkage in the concrete were reduced [15,33,37,38]. Macro PP fibres may be used to obtain the same level of reinforcement as that of steel, at a cost almost half of that of steel. Along with that, the labour cost was also reduced [39]. On the other hand, using Micro PP fibres in concrete enhances the durability of concrete while the shrinkage cracks are controlled [40]. The reinforcement of concrete with Micro PP fibres have a minimal effect on the concrete’s flexural and compressive strength [41].

This research intends to study the effect of the addition of PP fibres on the mechanical properties and durability of PAC having Silica fume as a partial replacement of cement (SPAC). First, an appropriate mix design for SPAC was decided. After that, SPAC was cast with three types of PP fibres, namely, Mono PP, Fibrillated PP, and Macro PP fibres, each with dosages of 1% and 1.5% (of the total volume of the mix); the optimum fibre type and dosage were determined based on highest compressive strength. The consequent concrete samples were tested for compressive strength, splitting tensile strength, mass, and compressive strength loss at 250 °C and the stress-strain curve was also obtained. A superplasticiser dosage of 1% (by weight of binder) was used throughout this study.

## 2. Materials and Methods

### 2.1. Materials

During this research, Cherat Cement was used, which is a Type I ordinary Portland cement (OPC), as per ASTM C 150-2007. The cement had a fineness value of 93.27% and a specific surface area of 267 m^2^/kg. The cement was obtained from a local supplier at College Road, Abbottabad, Pakistan. Silica fume was procured from Imporient Chemicals (PVT) limited, Islamabad, Pakistan. The specific surface area of the procured Silica fume was noted to be 14,000 m^2^/kg. An X-ray fluorescence test was carried out on the acquired sample of Silica fume in the ‘National centre of excellence in Geology’, University of Peshawar, Peshawar, Pakistan. The result of the XRF test is shown in Table 1.

**Figure 1:**
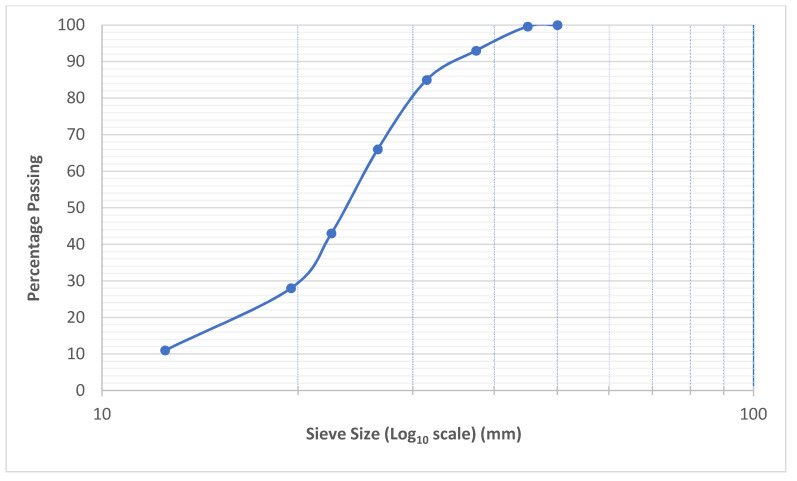
Gradation curve for the coarse aggregate.

**Figure 2:**
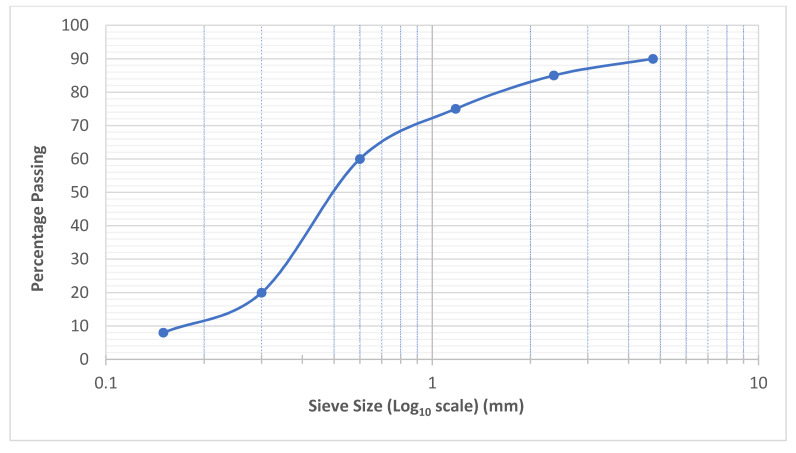
Gradation curve for fine aggregate.

Uncrushed river sand was used as the fine aggregate in this research. It was initially sieved through ASTM sieve number 14 before being used. The specific gravity of the fine aggregate was 2.71, with 0.68% water absorption and a fineness modulus of 2.62. The applied stresses are initially transferred to the coarse aggregate skeleton and then to the hardened grout in PAC. Thus, the use of appropriate shape, size, and quality of coarse aggregate need proper consideration [2]. ACI 304.1R-1997 recommends that the coarse aggregate used in PAC must first be washed to remove the dust on the surface to ensure better bonding with the injected grout. The coarse aggregate used was crushed granite with sizes ranging from 19.5 to 38 mm. The water absorption and specific gravity of the coarse aggregates used was 0.54% and 2.74, respectively. Both the fine aggregate and coarse aggregate were brought from a local quarry in Abbottabad. The gradation curves for coarse and fine aggregates are shown in Figure 1 and Figure 2, respectively. Chemrite AG-300, a 3rd generation superplasticiser, was used at a dosage of 1% to improve the flowability of the grout. Three types of polypropylene (PP) fibres, namely Mono, Macro, and Fibrillated PP fibres, were procured from Maxwell company, Karachi, Pakistan. The general characteristics of the procured PP fibres are mentioned in Table 2.

### 2.2. Mix Proportions

First, various mix designs, as shown in Table 3, were cast to determine the optimum mix design of preplaced aggregate concrete without any replacement or fibres (NPAC). Mix # 24 was selected as the optimum mix for NPAC. Afterwards, several mix designs were cast to determine the optimum mix design of the preplaced aggregate concrete with cement being partially substituted with Silica fume (SPAC), shown in Table 4. Mix # 4 was selected as the optimum mix for SPAC. Subsequently, different mix designs containing varying dosages of fibres were cast to determine the optimum mix design for the Polypropylene fibre-reinforced preplaced aggregate concrete with Silica fume partially substituting cement (FRPAC). The mix designs used are shown in Table 5. Mix # 17 was selected as the optimum mix for FRPAC. For determining the optimum mix designs of NPAC, SPAC, and FRPAC, the mix with the most suitable flowability and the maximum three-day compressive strength was used. Lastly, the final mix designs, as shown in Table 6, were cast, and the respective tests were performed on them. The optimum mix designs determined for NPAC were used as the starting mix design in the trials for determining the optimum mix design of SPAC. Likewise, the optimum mix design of SPAC was used as the initial mix design used for determining the optimum mix design of FRPAC. The mix design used for the final casting of NPAC, SPAC, and FRPAC was the optimum mix design determined for FPRAC.

**Figure 3:**
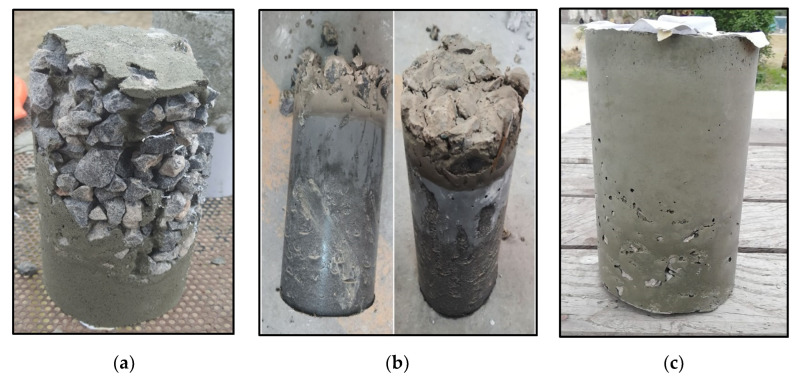
(**a**) Failed cast due to flowability being too low; (**b**) Failed cast due to flowability being too high, sand settled in the grout, inconsistent mix; (**c**) Sample with sufficient workability and acceptable cast.

A cast sample in which the grout was too thick is shown in Figure 3a, a sample in which the flowability of grout is too high is shown in Figure 3b, and a sample with sufficient workability is shown in Figure 3c.

### 2.3. Sample Preparation

**Figure 4:**
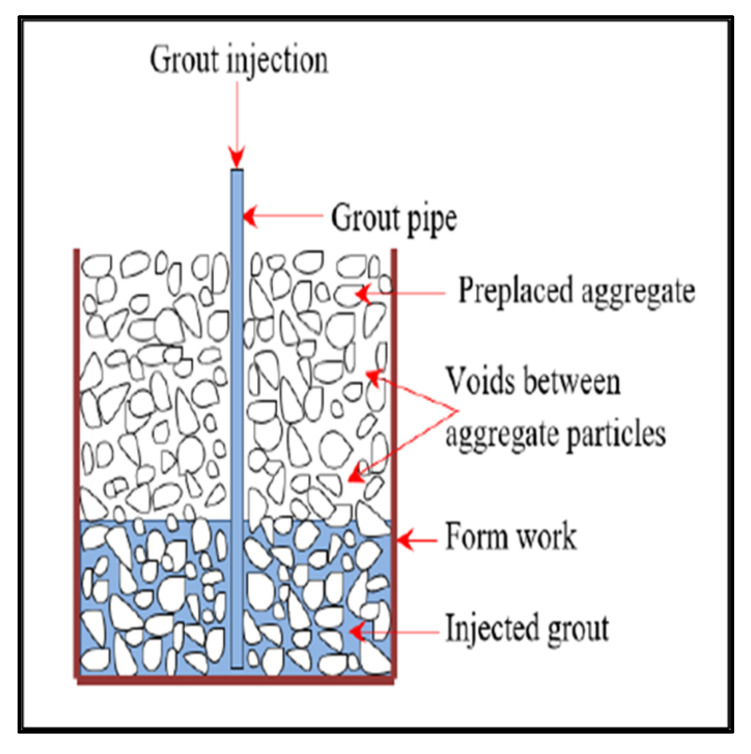
Mechanism of the pumping of grout [37].

The PAC samples were prepared in two steps. First, the coarse aggregate and PP fibres (in the case of FRPAC) were dry mixed for 2 min and then placed in the formwork. Secondly, the specialised grout was poured into the coarse aggregate matrix using a pipe and funnel, where the flow of grout was due to gravity (gravity method). In this research, cylindrical samples of 100 mm × 200 mm (diameter × height) were used; the cylindrical moulds had a flat base with an opening at the top, from where the aggregates were placed, and the grout was injected. Figure 4 illustrates the mechanism of the pumping of the grout in the preplaced aggregate concrete [42].

The grout, a mixture of cement, Silica fume (in case of SPAC and FRPAC), fine aggregate, water, and superplasticiser, were prepared in an electric mixer. The contents were initially dry mixed for 3 min and then wet mixed for 5 min, with the electric mixer set at high revolutionary speeds to reduce the settlement of sand in the mix and to obtain a uniform and consistent mix. For the gravity method, a pipe of 40 mm diameter was used through which the grout was injected into the cylindrical moulds, as shown in Figure 4. With the pipe inserted in the cylindrical moulds, to the bottom of the mould, a funnel was attached to the top of the pipe through which the grout was slowly poured into the pipe. As the grout was being poured into the top, the pipe was simultaneously removed very slowly. This was performed so that the grout may spread evenly throughout the height of the sample and also to ensure that the pipe does not get choked with the grout. During the injection of grout, special care was taken to ensure that the grout did not leak out of the mould. After the casting was completed, the top surface was finished off to get a smooth surface and then the cast samples were cured at room temperature for 24 h. Subsequently, at the end of the 24 h, the samples were demoulded and then placed in a water tank for a maximum of 28 days.

### 2.4. Testing Methods

**Figure 5:**
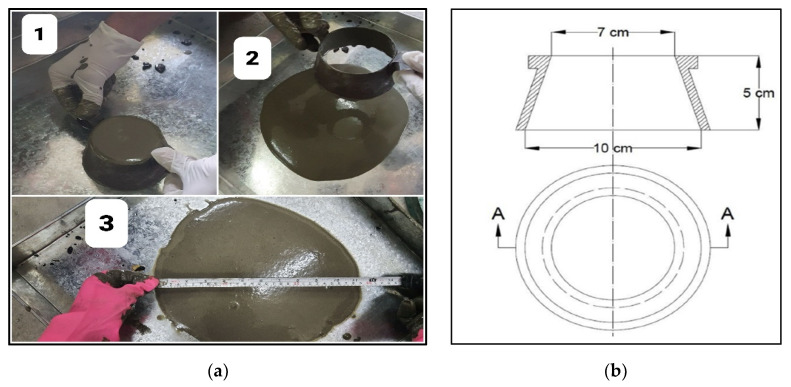
(**a**) Method for testing flowability of grout; (**b**) Dimensions of conical mould used for grout flowability measurement [38].

The flowability of the prepared grout was tested using a flow table and conical mould [1,43,44], as shown in Figure 5a. The conical mould was filled with the grout [Step 1 in Figure 5a], then the mould was quickly lifted [Step 2 in Figure 5a], and the spread of the grout was measured using a measuring tape [Step 3 in Figure 5a]. The dimensions of the mould are illustrated in Figure 5b [43].

Cylindrical samples of 100 mm × 200 mm were prepared, cast, and tested as per ASTM C39M-18, and ASTM C496M-17, for compressive strength and splitting tensile strength, respectively. The 100 mm × 200 mm samples, at 28 days of curing, were placed in a Universal Testing Machine (UTM), at COMSATS University Islamabad, Abbottabad campus, Pakistan, to determine the stress-strain relationship of the samples.

The cylindrical samples, with 100 mm diameter and 200 mm height, at 28 days of curing were initially weighed and then placed in an oven at a temperature of 250 °C for 24 h. Finally, after 24 h, the samples were again weighed, and the mass difference was recorded. The samples were also tested for compressive strength, and the strength loss due to high temperature was determined.

## 3. Results and Discussion

### 3.1. Flowability of Grout

In PAC, sufficient flowability of the grout is crucial for the successful casting of the concrete. Otherwise, the grout might not penetrate some voids, subsequently leading to honeycombing. Yousefi Oderji et al. used a conical mould to determine the flowability of the grout [38]. The same method was followed in this study, as shown in Section 2.4, to determine the flowability of the prepared grout. It was found that for our 100 mm × 200 mm moulds, a flowability of approximately 280 mm was found to be the optimum value for NPAC and SPAC, as it led to a more acceptable casting of those mixes. However, when PP fibres were added along with the coarse aggregate into the mould, honeycombing was witnessed in the cast samples. Thus, trial and error established that for FRPAC samples, a grout flowability of 356 mm was the optimum value. Assessing the effect of Silica fume on the flowability of the grout, it is evident from Table 4, that higher Silica fume contents led to lower flowability values. Thus, greater water-to-binder (w/b) ratios were required to obtain optimum flowability, with the other parameters being the same. From Table 5, it is evident that higher w/b ratios were required for the successful casting of the mix. It may be because the addition of PP fibres in the mould impeded the spread of grout in the fibre and coarse aggregate skeleton. Thus, higher flowability was required, which was achieved by increasing the w/b ratio.

### 3.2. Compressive Strength

**Figure 6:**
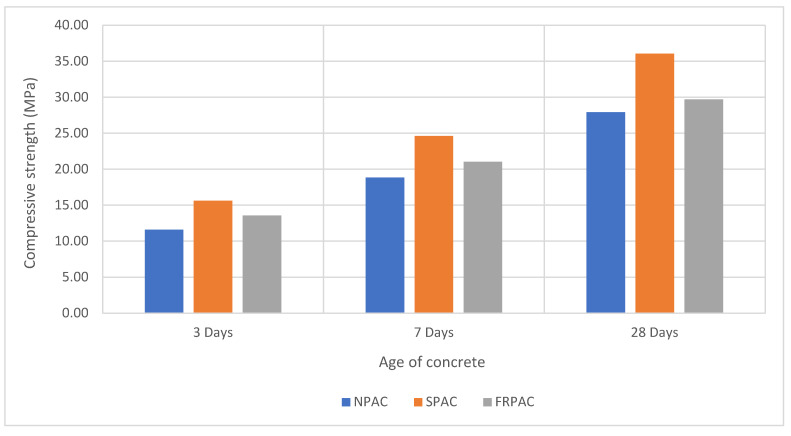
Compressive strength of cast samples vs. age of concrete.

The results of the compressive strength of NPAC, SPAC, and FRPAC are displayed in Figure 6. Partially replacing cement with Silica fume improved the compressive strength of the cast samples. The increase in compressive strength can be attributed to the higher pozzolanic activity of silica fume. Along with that, the high fineness of Silica fume strengthens the microstructure by improving the particle packing density [14]. It is evident from Figure 6 that the inclusion of PP fibres into SPAC decreased the compressive strength. The strength loss at 28 days of curing was 17.61% compared to SPAC. The loss in compressive strength might be due to the existence of cavities in the matrix produced as a result of the addition of fibres in the mix. Increasing fibre content leads to balling effect, clustering, and the formation of voids, subsequently decreasing the concrete’s strength while also making it more susceptible to cracking. The presence of fibres impedes the spread of grout in the coarse aggregate skeleton, thus reducing compressive strength [45].

### 3.3. Splitting Tensile Strength

**Figure 7:**
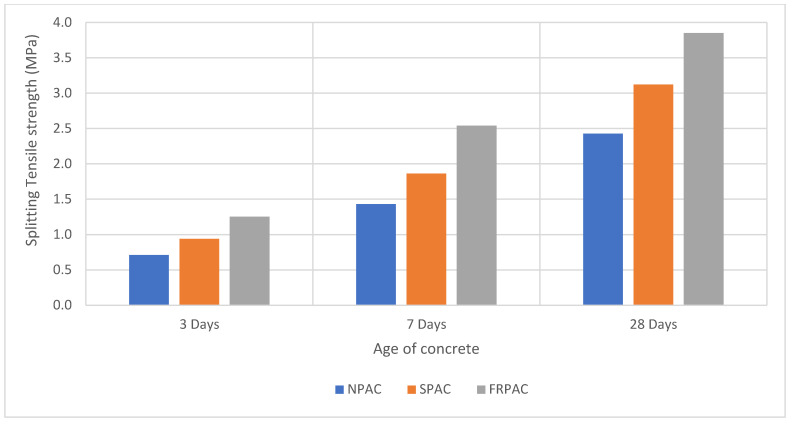
Splitting tensile strength of cast samples vs. age of concrete.

The splitting tensile strength test was conducted on 100 mm × 200 mm cylindrical samples. The results of those tests are shown in Figure 7. As compared to NPAC, SPAC is seen to have a greater splitting tensile strength; the strength difference was 22.3% at 28 days. As evident from Figure 7, the addition of PP fibres led to an increase in the splitting tensile strength of the cast samples. FRPAC had 37% and 18.9% higher splitting tensile strength values than NPAC and SPAC at the age of 28 days, respectively. This improvement in the tensile strength of FRPAC samples, under indirect tension, may be due to the crack arrestment property of the fibres. Greater dosages of PP fibres restricts the formation of cracks in the cast samples; therefore, enhancing the splitting tensile strength of FRPAC [8].

### 3.4. Mass Loss and Compressive Strength Loss at 250 °C

**Figure 8:**
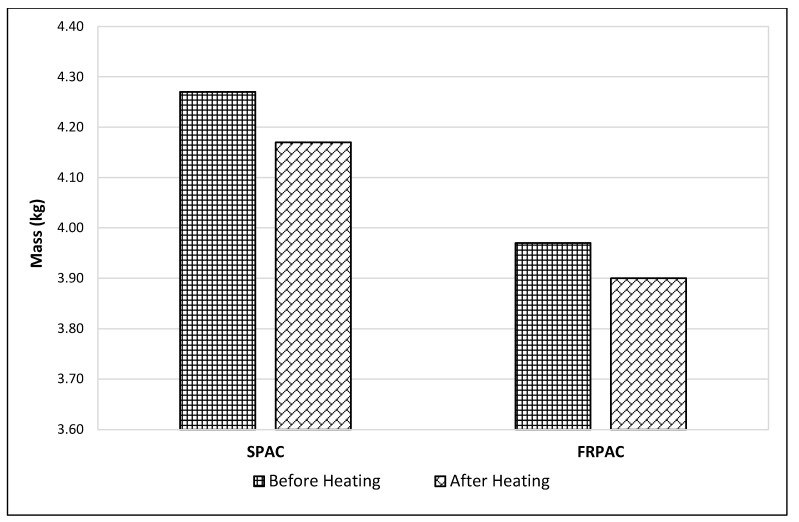
Mass loss at 250 °C.

The FRPAC samples at 28 days of curing were air-dried and then weighed. Afterwards, the samples were placed in an oven at a temperature of 250 ± 5 °C, for 24 h. After 24 h, these samples were taken out of the oven and then, after cooling, were weighed again. The mass loss was calculated by subtracting the mass after heating from the mass before being placed in the oven. It was observed that the mass loss in SPAC samples was 30% greater than that of FRPAC samples, as shown in Figure 8.

**Figure 9:**
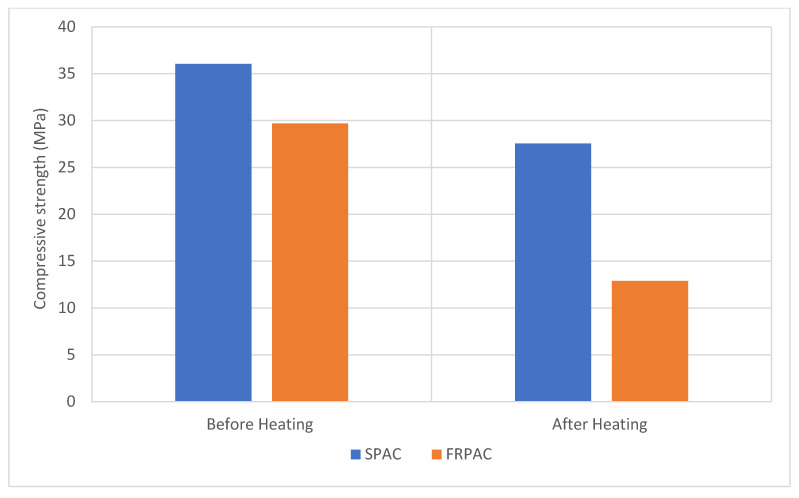
Compressive strength loss at 250 °C.

The aforementioned samples were then placed in a compression testing machine to determine their post-heating compressive strength. Both FRPAC and SPAC experienced a loss in compressive strength after heating. However, FRPAC samples underwent a substantial loss in compressive strength, approximately 56.6%, compared to just 23.6% in SPAC samples. The results of compressive strength both before and after heating are illustrated in Figure 9. The greater strength loss might be due to the melting of PP fibres at 160 to 170 °C, which leads to microcracks and greater porosity, thus decreasing the strength of concrete [46].

### 3.5. Stress-Strain Relationship

**Figure 10:**
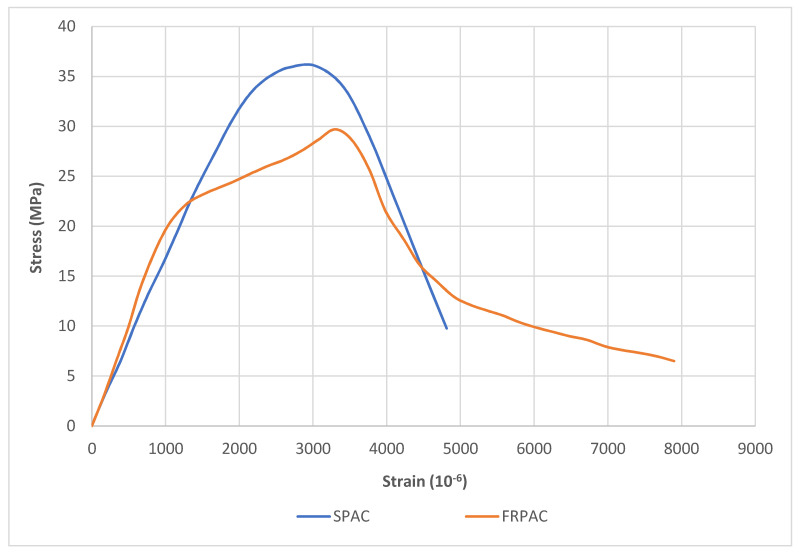
Stress-strain relationship.

FRPAC and SPAC samples, after 28 days of curing, were placed in a UTM to determine the stress-strain relationship of both mixes. Three samples each for SPAC and FRPAC were tested. Due to the overlapping of the curves, only one of the curves for each SPAC and FRPAC was used in Figure 10. As illustrated in Figure 10, FRPAC achieved greater strain values for given applied stress compared to SPAC. Thus, referring to the stress-strain plot, it can be said that the addition of PP fibres in the mix leads to an enhancement in the ductility of the concrete.

## 4. Conclusions

This study was undertaken to observe the influence of the inclusion of Polypropylene (PP) fibres in preplaced aggregate concrete (PAC) with cement being partially replaced by Silica fume. PAC without Silica fume or PP fibres (NPAC), PAC with Silica fume as supplementary cementitious material (SPAC), and PP fibre-reinforced SPAC (FRPAC) were tested at 3, 7, and 28 days of curing. Compressive strength test, splitting tensile strength test, mass loss, and compressive strength test at 250 °C were performed on the samples. After thoroughly analysing the results, it was concluded that:

- SPAC had higher compressive strength than NPAC; the difference in strength was noted to be 22.6% at 28 days. This may be attributed to higher pozzolanic activity and high sample density due to the addition of Silica fume.
- The inclusion of fibres in SPAC led to a reduction in the compressive strength of the cast samples. At 28 days of curing, FRPAC had 17.61% lower compressive strength than SPAC. The loss in compressive strength might be due to the generation of a greater number of voids in the cast samples due to the addition of fibres. Furthermore, higher fibre dosages inhibit the flow of grout in the coarse aggregate skeleton.
- Overall, FRPAC had the highest splitting tensile strength among the three mixes. At the age of 28 days, FRPAC had 37% and 18.9% higher splitting tensile strength values than NPAC and SPAC, respectively. It is evident that the inclusion of PP fibres enhances the deformation capacity of preplaced aggregate concrete. It may be mainly due to the bridging action of the fibres, preventing the formation and spread of microcracks.
- The mass loss in SPAC at 250 °C was 30% greater than that of FRPAC. SPAC suffered a mass loss of 2.34% as compared to 1.76% in FRPAC.
- The compressive strength loss at 250 °C was considerably greater in FRPAC compared to SPAC. SPAC suffered a compressive strength loss of 23.6% compared to 56.6% in FRPAC.
- Stress-strain curves of FRPAC and SPAC showed that FRPAC exhibited greater strain for the amount of stress applied. Hence it could be deduced from these curves that the inclusion of Polypropylene fibres imparted ductile properties in concrete.

## Figures and Tables

**Table 1:** Chemical composition of Silica fume used.

Chemical Composition	Percentage	ASTM C618 Requirement
SiO_2_	97.323	SiO_2_ + Fe_2_O_3_ + Al_2_O_3_ > 70%
Fe_2_O_3_	0.044	
Al_2_O_3_	0.003	
SO_3_	2.043	-
K_2_O	0.545	-
MnO	0.016	-
CuO	0.015	-

**Table 2:** General characteristics of the procured Polypropylene fibres.

Property	Value	Standard Used
Compressive strength (MPa)	38–55	ASTM D695
Flexural strength (MPa)	41–56	ASTM D790
Tensile strength at break (MPa)	31–42	ASTM D638
Elongation at break (%)	100–600	ASTM D638
Water absorption (%)	Negligible (0.01–0.03)	ASTM D570
Specific Gravity	0.90–0.91	ASTM D792
Ignition point	593 °C	-
Melting Point	160–170 °C	-
Heat and UV stabilisation	Long Term	-
Thermal Conductivity	2.8 × 10^−4^ Cal cm/s cm^2^ °C	ASTM C177
Tensile Modulus (MPa)	1140–1560	ASTM D638
Compressive modulus (MPa)	1030–2070	ASTM D695
Flexural Modulus (MPa @ 25 °C)	1170–1730	ASTM D790
Rockwell hardness	R80–R102	ASTM D785
Electrical Conductivity	Low	-
Salt Resistance	High	-
Acid Resistance	High	-
Alkali Resistance	100% (alkali proof)	-

**Table 3:** Mix designs used to determine optimum mix design for NPAC.

S. No	S/B	W/B	SP Dosage (% by Weight of Binder)	SP Model	Flowability (mm)	Comments	Three-Day Compressive Strength (MPa)
1	0.5	0.4	2	Chemrite AG 300	712	Flowability of grout too high	-
2	0.5	0.4	1	AG 300	585	Flowability of grout too high	-
3	0.5	0.37	0	AG 300	102	Grout too thick	-
4	0.5	0.37	0.1	AG 300	114	Grout too thick	-
5	0.5	0.37	0.15	AG 300	120	Grout too thick	-
6	0.5	0.37	0.175	AG 300	123	Grout too thick	-
7	0.5	0.37	0.2	AG 300	127	Grout too thick	-
8	0.5	0.37	0.225	AG 300	153	Grout too thick	-
9	0.5	0.37	0.25	AG 300	178	Grout too thick	-
10	0.5	0.37	0.275	AG 300	204	Grout too thick	-
11	0.5	0.37	0.3	AG 300	229	Grout too thick	-
12	0.5	0.37	0.325	AG 300	254	Grout too thick	-
13	0.5	0.37	0.35	AG 300	305	Sufficient flowability of grout	13.75
14	0.5	0.37	0.375	AG 300	356	Flowability of grout too high	-
15	0.5	0.37	0.4	AG 300	394	Flowability of grout too high	-
16	0.5	0.37	0.425	AG 300	432	Flowability of grout too high	-
17	0.5	0.37	0.45	AG 300	483	Flowability of grout too high	-
18	0.5	0.37	0.475	AG 300	508	Flowability of grout too high	-
19	0.5	0.37	0.5	AG 300	534	Flowability of grout too high	-
20	1	0.37	0.35	AG 300	178	Grout too thick	-
21	1	0.37	0.5	AG 300	204	Grout too thick	-
22	1	0.37	0.75	AG 300	229	Grout too thick	-
23	1	0.37	0.8	AG 300	242	Grout too thick	-
**24**	**1**	**0.37**	**1**	**AG 300**	**280**	**Sufficient flowability**	**16.23**
25	1.5	0.37	0.35	AG 300	115	Grout too thick	-
26	2	0.37	2	AG 300	102	Grout too thick	-
27	2	0.4	2	AG 300	140	Grout too thick	-
28	2	0.45	2	AG 300	191	Grout too thick	-
29	2	0.47	2	AG 300	216	Grout too thick	-
30	2	0.5	2	AG 300	254	Grout too thick	-

**Table 4:** Mix designs used to determine optimum mix design for SPAC.

S. No	S/B	W/B	Silica Fume (%Age by Weight of Binder)	SP dosage (% by Weight of Binder)	SP Model	Flowability (mm)	Comments	Three-Day Compressive Strength (MPa)
1	1	0.37	5	1	AG 300	280	Sufficient flowability	17.87
2	1	0.37	10	1	AG 300	229	Grout too thick	-
3	1	0.38	10	1	AG 300	254	Grout too thick	-
**4**	**1**	**0.39**	**10**	**1**	**AG 300**	**280**	**Sufficient flowability**	**19.73**
5	1	0.39	15	1	AG 300	242	Grout too thick	-
6	1	0.41	15	1	AG 300	267	Grout too thick	-
**7**	1	0.41	20	1	AG 300	166	Grout too thick	-
8	1	0.45	20	1	AG 300	254	Grout too thick	-

**Table 5:** Mix designs used to determine optimum mix design for FRPAC.

S. No	S/B	W/B	Silica Fume (%Age by Weight of Binder)	SP Dosage (% by Weight of Binder)	Fibre Type	Fibre Content (% by the Total Volume of Mould)	Flowability (mm)	Compressive Strength (MPa)
1	1	0.39	10	1	Mono	1	280	-
2	1	0.39	10	1	Mono	1.5	280	-
3	1	0.45	10	1	Mono	1	294	-
4	1	0.45	10	1	Mono	1.5	294	-
5	1	0.45	10	1	Macro	1	294	-
6	1	0.45	10	1	Macro	1.5	294	-
7	1	0.45	10	1	Fibrillated	1	294	-
8	1	0.45	10	1	Fibrillated	1.5	294	-
9	1	0.5	10	1	Mono	1.5	311	-
10	1	0.55	10	1	Mono	1.5	332	-
11	1	0.65	10	1	Mono	1.5	375	-
12	1	0.7	10	1	Mono	1.5	388	-
13	1	0.8	10	1	Mono	1.5	409	-
14	1	0.6	10	1	Mono	1	356	11.1
15	1	0.6	10	1	Mono	1.5	356	9.78
16	1	0.6	10	1	Macro	1	356	9.96
**17**	**1**	**0.6**	**10**	**1**	**Macro**	**1.5**	**356**	**13.56**
18	1	0.6	10	1	Fibrillated	1	356	13.32
19	1	0.6	10	1	Fibrillated	1.5	356	11.85

**Table 6:** Mix designs used for the final casting.

Name	S/B	W/B	Silica Fume (%Age by Weight of Binder)	SP Dosage (% by Weight of Binder)	Fibre Type	Fibre Content (% by the Total Volume of Mould)
NPAC	1	0.6	0	1	-	-
SPAC	1	0.6	10	1	-	-
FRPAC	1	0.6	10	1	Macro	1.5

## Data Availability

The data used in this research was collected from published literature.
